# Variants on the promoter region of *PTEN *affect breast cancer progression and patient survival

**DOI:** 10.1186/bcr3076

**Published:** 2011-12-15

**Authors:** Tuomas Heikkinen, Dario Greco, Liisa M Pelttari, Johanna Tommiska, Pia Vahteristo, Päivi Heikkilä, Carl Blomqvist, Kristiina Aittomäki, Heli Nevanlinna

**Affiliations:** 1 Department of Obstetrics and Gynecology, Helsinki University Central Hospital, P.O. Box 700, 00029 HUS, Helsinki, Finland; 2 Department of Pathology, Helsinki University Central Hospital, P.O. Box 400, 00029 HUS, Helsinki, Finland; 3 Department of Oncology, Helsinki University Central Hospital, P.O. Box 180, 00029 HUS, Helsinki, Finland; 4 Department of Clinical Genetics, Helsinki University Central Hospital, P.O. Box 140, 00029 HUS, Helsinki, Finland

## Abstract

**Introduction:**

The *PTEN *gene, a regulator of the phosphatidylinositol-3-kinase (PI3K)/Akt oncogenic pathway, is mutated in various cancers and its expression has been associated with tumor progression in a dose-dependent fashion. We investigated the effect of germline variation in the promoter region of the *PTEN *gene on clinical characteristics and survival in breast cancer.

**Methods:**

We screened the promoter region of the *PTEN *gene for germline variation in 330 familial breast cancer cases and further determined the genotypes of three detected *PTEN *promoter polymorphisms -903GA, -975GC, and -1026CA in a total of 2,412 breast cancer patients to evaluate the effects of the variants on tumor characteristics and disease outcome. We compared the gene expression profiles in breast cancers of 10 variant carriers and 10 matched non-carriers and performed further survival analyses based on the differentially expressed genes.

**Results:**

All three promoter variants associated with worse prognosis. The Cox's regression hazard ratio for 10-year breast cancer specific survival in multivariate analysis was 2.01 (95% CI 1.17 to 3.46) *P *= 0.0119, and for 5-year breast cancer death or distant metastasis free survival 1.79 (95% CI 1.03 to 3.11) *P *= 0.0381 for the variant carriers, indicating *PTEN *promoter variants as an independent prognostic factor. The breast tumors from the promoter variant carriers exhibited a similar gene expression signature of 160 differentially expressed genes compared to matched non-carrier tumors. The signature further stratified patients into two groups with different recurrence free survival in independent breast cancer gene expression data sets.

**Conclusions:**

Inherited variation in the *PTEN *promoter region affects the tumor progression and gene expression profile in breast cancer. Further studies are warranted to establish *PTEN *promoter variants as clinical markers for prognosis in breast cancer.

## Introduction

Hereditary predisposition to breast cancer is caused by variation in multiple genes affecting the cancer risk with varying penetrance. Mutations in the main high penetrance genes *BRCA1 *and *BRCA2 *are mostly found in families with multiple breast cancer cases particularly with early onset and with ovarian cancer [1,2], and may also affect breast cancer survival among the mutation carriers [3,4]. Strong familial breast cancer predisposition is also present in rare cancer syndromes. Rare germline mutations in the *TP53 *gene cause Li-Fraumeni syndrome with highly increased risk for various malignancies, including breast cancer [5]; whereas a common *TP53 *variant in the population, R72P with functional effect on p53 protein, has been shown to affect breast cancer survival [6,7]. Another rare cancer syndrome with increased breast cancer risk is Cowden syndrome caused by germline mutations in the *PTEN *gene [8,9]. Patients with Cowden syndrome develop multiple hamartomatous, mostly benign neoplasms especially on the skin and mucous membrane, and also have a lifetime risk of 25 to 50% for breast cancer and an increased risk of developing epithelial thyroid and endometrial carcinomas [10]. *PTEN *mutations causing Cowden syndrome include a noticeable number of variants on the promoter region affecting transcriptional levels of the gene or causing abnormal translation of the protein [11,12]. The promoter of *PTEN *has been characterized in the 5' region of the gene between nucleotides -1344 and -747 from translation start site and it contains binding sites, for example, for p53 and Sp1 transcription factors [12-14]. So far, *PTEN *germline variation increasing susceptibility to cancer outside Cowden syndrome, or associating with tumor progression, has not been detected [15-17].

The *PTEN *(*Phosphatase and tensin homolog*) gene is a tumor suppressor gene located on chromosome 10q23 and is mutated in multiple cancers [18,19]. The PTEN protein, a dual specificity phosphatase with lipid and protein phosphatase activities, functions as a negative regulator of PI3K/Akt oncogenic pathway [20]. Alterations in this pathway are among the most common changes in human carcinogenesis [21]. In addition to the PI3K/Akt pathway regulation, when localized to the nucleus, PTEN takes part, for instance, in regulation of chromosomal integrity, acetylation of p53, DNA-damage response and the induction of apoptosis [22]. In breast tumors, PTEN expression is often lost through mutations or epigenetic mechanisms [23,24]. Reduced PTEN expression [24-26] and the dysregulated PI3K/Akt pathway [27,28] have been associated with aggressive breast cancer phenotype and poor outcome of the disease. Breast tumors originating by dysfunctional *BRCA1 *often suffer PTEN loss through gross mutations [29]. Furthermore, tumors with reduced PTEN protein expression have been shown to carry a particular gene expression signature that predicts worse outcome and metastasis in breast cancer as well as in prostate and bladder carcinomas [30]. Recently, a moderate decrease in *PTEN *expression to 80% of the normal level has been shown to increase susceptibility to develop cancer in mice, particularly in mammary tissue [31].

To investigate the role of potentially regulatory *PTEN *germline genetic variation on clinical characteristics and survival in breast cancer, we screened the promoter region of *PTEN *from 330 familial breast cancer cases. We genotyped the detected promoter variants in a large set of familial and unselected breast cancer patients to evaluate the effects of the variants on tumor phenotype and disease outcome. We also compared the gene expression profiles in breast cancer tumors of the variant carriers and non-carriers, with further survival analyses on the differentially expressed genes in breast cancer gene expression data sets.

## Materials and methods

### Subjects

The promoter region of the *PTEN *gene was screened for germline variation in 330 patients from families with multiple cases of breast or ovarian cancer, found negative for *BRCA1 *and *BRCA2 *mutations by screening the coding regions of the genes using denaturing gradient gel electrophoresis (DGGE) and for the large exon 11 of *BRCA1 *and exons 10 and 11 of *BRCA2 *using protein truncation test (PTT).

Altogether 1,870 unselected breast cancer patients and 542 additional familial cases were included in the genotyping of the promoter variants. The unselected cohort consisted of two series of patients, collected in Helsinki University Central Hospital's Department of Oncology in 1997 to 1998 and 2000 (884 patients) [32,33], and on the Department of Surgery in 2001 to 2004 (986 patients) [34] covering 79% and 87% of all consecutive newly diagnosed breast cancer patients, respectively. The familial breast cancer cases were collected at Helsinki University Central Hospital's Department of Clinical Genetics. The familial series consisted of patients with strong familial background with three or more breast or ovarian cancers among first or second degree relatives, including the proband, and of familial cases with one first degree relative and the proband affected with breast cancer. The *BRCA1 *and *BRCA2 *mutations were excluded as described earlier [35].

The cancer diagnoses were confirmed through the Finnish Cancer Registry and hospital records. Information on death due to breast cancer was obtained from the Finnish Cancer Registry, which collects diagnostic as well as death information on all cancer patients in Finland, and the distant metastasis data were retrieved from hospital records collected with routine follow-up investigations of breast cancer patients for five years from diagnosis. The survival was calculated as the time from the diagnosis of the first invasive breast cancer to the date of death due to breast cancer or of diagnosis of a distant metastasis. This study was performed with informed consent from the patients as well as permission from the Ethics Committee of the Helsinki University Central Hospital and from the Ministry of Social Affairs and Health in Finland.

Information on tumor histology, grade, size, nodal status and distant metastases at diagnosis were collected from pathology reports [36]. Additionally, a breast cancer pathologist re-reviewed 1,423 tumors (56% of all) for tumor histology and grade, according to Scarff-Bloom-Richardson modified by Elston and Ellis [37]. Estrogen receptor (ER) and progesterone receptor (PR) status were retrieved from pathology reports [36]. For both ER and PR status, samples with > 10% of the cancer cells stained positive with immunohistochemistry were considered as positive. HER2 status was based on immunohistochemical staining (samples with < 10% of the cells stained were considered negative and > 90% positive) and gene amplification with chromogenic *in situ *hybridization (CISH; over six replications was considered positive and zero to five replications was considered negative) on tumor microarrays as described in [38]. p53 protein expression was evaluated by immunohistochemical staining as previously described [6]. Samples were defined as positive for p53 when more than 20% of the cancer cells were positive for the staining. Ki67 status was defined as described [39] with strong positive (3) expression considered when ≥ 30%, intermediate (2) when 20 to 29%, weak positive (1) when 5 to 19%, and negative (0) when < 5% of the cancer cells were stained with Ki67 antibody. Altogether 2,401 invasive breast tumors from 2,256 patients were taken into the analysis of the variants' association with tumor characteristics.

A total of 2,204 patients with invasive breast cancer and follow-up data available were included in the survival analysis. The median follow-up time in 10-year breast cancer specific analysis was 83 months, and for 5-year breast cancer death or distant metastasis free survival (BDDM) was 47 months. Of all patients in the survival analysis, 298 died from breast cancer within 10 years from diagnosis, and 352 developed distant metastasis or died of breast cancer within 5 years from diagnosis.

### Mutation screening and genotyping

The complete promoter region between nucleotides -1344 and -747 [12] of the *PTEN *gene were screened for variation on genomic DNA isolated from blood samples of 330 familial breast cancer patients using conformation sensitive gel electrophoresis (CSGE) heteroduplex analysis. All variants were verified by bidirectional sequencing.

CSGE was further used to determine the genotypes of the variants -903GA and -975GC, which were located in the same CSGE amplicon (covering nucleotides from -617 to -1087) in the complete data set. All samples showing different banding patterns on gel were verified by sequencing. The -1026CA variant was genotyped using Sequenom i-PLEX with service provided by the Finnish Genome Center. Of the 2,412 patients genotyped, a successful result was obtained from 2,375 (98.5%) individuals for -1026CA and from 2,369 (98.2%) individuals for -903GA and -975GC variants.

### Gene expression analysis

Total RNA was extracted from primary breast tumors of 183 patients, including 10 cases carrying a promoter variant. Of them, 151 were collected as a part of the unselected series and 32 patients belonged to additional familial breast cancer cases. The samples were processed and hybridized to Illumina HumanHT-12 v3 Expression BeadChips (Illumina Inc, San Diego, CA, USA) containing 24,660 Entrez Gene entities, according to the manufacturer's recommendations (http://www.illumina.com).

Microarray raw data were imported into R v2.11 (http://cran.r-project.org) and processed by the methods included in the BioConductor facilities [40]. Briefly, after quality control [41], the data were normalized using the quantile method [42] and the gene expression matrix from the tumors of 10 *PTEN *promoter variant carriers and 10 wild type controls matched with tumor histology, estrogen and progesterone receptor status, HER2 overexpression/amplification, grade, tumor size, p53 status and Ki67 expression was obtained by averaging the probes mapping to the same Entrez Gene IDs (http://www.ncbi.nlm.nih.gov/Entrez). Differential expression between the tumors of promoter variant carriers and non-carriers was assayed by moderated t-test. Genes with *P *< 0.01 were considered to be significant [43] and further analyzed. Functional annotation was performed on the differentially expressed gene list using the DAVID functional annotation tools (http://david.abcc.ncifcrf.gov/) [44]. The categories with Fisher's exact test *P*-value < 0.05 were considered to be significantly enriched.

The 160 differentially expressed genes were further used to cluster the patients of the whole data set and three publicly available breast cancer gene expression series into two groups by an unsupervised clustering method. The K-means algorithm was iterated 100,000 times to ensure maximum reliability and the results were stabilized by imposing a pre-defined random number selection algorithm at the beginning of the process.

### Statistical analyses

Statistical analyses were performed using SPSS 15.0 software (SPSS, Inc., Chicago, IL, USA). Pearson's χ^2 ^test was used to evaluate the association of different genotypes on tumor characteristics. Fischer's exact test was applied when the expected number of cell count was less than five. All *P*-values were two-sided. The effects of the variants on the prognosis of the patients were analyzed using Kaplan-Maier survival plots with Log-Rank test. The survival analyses were performed on long-term breast cancer specific 10-year follow-up and on short term combined analysis on breast cancer death or distant metastasis (BDDM) with 5-year follow-up. The survival hazard ratios were calculated using univariate Cox's regression analysis. Independence of the variants in relation to common prognostic factors (tumor size, nodal status, primary metastasis, estrogen receptor, progesterone receptor, Her2, p53, Ki67, grade) was evaluated by constructing Cox's multivariate models with SPSS 15.0 backward conditional algorithm. All variables were set as categorical. For increased statistical power, the three promoter variants were combined for the multivariate analysis.

## Results

### Variant discovery

Two previously known polymorphic variants (-1026CA/rs34149102 and -903GA/rs1044322) and one novel variant (-975GC) were observed in the screening of the 330 familial breast cancer patients. The promoter variant numbering as nucleotides from translation start site is matched with the numbering used by Zhou *et al. *[12]. In the complete data set of 1,870 unselected and 542 additional familial breast cancer cases the -1026CA genotype was present in 2%, -975GC in 1%, and -903GA in 3% of the patients. Two patients were also found with both -1026CA and -903GA, and one patient with -903GA and -975GC variants.

### *PTEN *promoter variants associate with markers of aggressive disease and survival of breast cancer patients

To evaluate the possible associations of the three recurrent *PTEN *promoter variants detected in the patient samples (-903GA, -975GC, and -1026CA) with the tumor phenotype, we calculated the correlations between the *PTEN *promoter variant status and tumor characteristics (tumor size, lymph node and distant metastasis at diagnosis, tumor histology, grade, estrogen and progesterone receptor status, HER2 over-expression, p53 status, and Ki67 proliferation marker expression) (Additional file 1). The -975GC variant was found to associate with a significantly higher frequency with distant metastasis at diagnosis (OR 4.99, 95% CI 1.69 to 14.78; *P *(Fischer) = 0.013) and the -1026CA variant with high expression of Ki67 (OR 2.21; 95% CI 1.15 to 4.28; *P *= 0.015).

**Figure 1:**
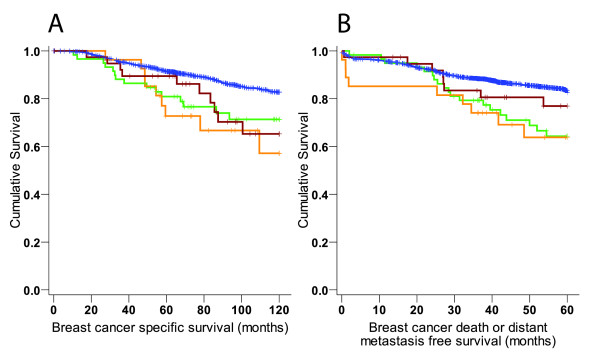
**Kaplan-Meier plots of cumulative survival for breast cancer patients carrying a *PTEN *promoter variant**. The plots for patients with different variants are shown for -903GA in green, -975GC in orange, -1026CA in red, and for non-carrier Wild type in blue. All variants showed significant long term survival effect in breast cancer specific 10-year analyses **(A) **with cumulative survival at 120 months of 82.7% (95% CI 80.7 to 84.7%) for non-carriers, 71.3% (95% CI 58.8 to 83.8%) for -903GA (*P *= 0.016), 57.2% (95% CI 33.1 to 81.3%) for -975GC (*P *= 0.002), and 65.3% (95% CI 47.1 to 83.5%) for -1026CA (*P *= 0.014). Two variants also showed significant effect in five year breast cancer death or distant metastasis free analysis **(B) **with cumulative survival at 60 months of 82.5% (95% CI 80.7 to 84.3%) for non-carriers, 64.3% (95% CI 51.2 to 77.3%) for -903GA (*P *= 0.002), 63.8% (95% CI 44.4 to 83.2%) for -975GC (*P *= 0.010), and 76.9% (95% CI 62.6 to 91.0%) for -1026CA (*P *= 0.279).

The effects of the variants on disease outcome were evaluated for 10-year breast cancer specific survival and 5-year BDDM free survival by Kaplan-Meier estimates with LogRank test and Cox's regression analyses. All three promoter variants were found to be significantly associated with worse long term survival and variants -903GA and -975GC also with short term breast cancer death or distant metastasis free survival (Figure 1). Patients with -903GA, -975GC or -1026CA variant had 10-year breast cancer specific survival rates of 71% (*P *= 0.016), 57% (*P *= 0.002), and 65% (*P *= 0.014) respectively, compared to survival rate of 83% of non- carriers (Figure 1A). In the five-year analysis, the cumulative survival for breast cancer specific death or distant metastasis was 64% for -903GA carriers (*P *= 0.002), 64% for -975GC carriers (*P *= 0.010), and 77% for -1026CA carriers (*P *= 0.279) compared to 83% for the non-carriers, with the difference for the first two variants being statistically significant (Figure 1B). The univariate Cox's regression analyses concurred with the results of the Kaplan-Meier analysis (Table 1), showing that harboring any of the three variants affected the 10-year breast cancer specific survival with a hazard ratio of 2.17 (*P *= 0.00002) and the 5-year BDDM with a hazard ratio of 1.97 (*P *= 0.00011). The multivariate Cox's regression models adjusted with conventional prognostic markers demonstrate that carrying any of the three promoter variants is an independent prognostic factor, with approximately two-fold increased risk of death or distant metastasis for the carriers (Table 2).

**Table 1:** Univariate Cox regression survival analysis of breast cancer patients with *PTEN *promoter variants.

	10-year breast cancer specific			5-year BDDM		
**Variant**	***P*-value**	**HR**	**95% CI**	***P*-value**	**HR**	**95% CI**
-903GA	0.01521	1.90	1.13 to 3.20	0.00201	2.07	1.31 to 3.29
-975GC	0.00357	2.68	1.38 to 5.21	0.01220	2.33	1.20 to 4.52
-1026CA	0.01829	2.06	1.13 to 3.77	0.28165	1.44	0.74 to 2.79
any	0.00002	2.17	1.52 to 3.10	0.00011	1.97	1.40 to 2.79

The 10-year breast cancer-specific survival and the 5-year breast cancer death or distant metastasis (BDDM) free survival analyses show significant association with reduced survival for all three *PTEN *promoter variants.

**Table 2:** Multivariate Cox regression survival analysis of the *PTEN *promoter variants, adjusted for common prognostic factors.

10-year breast cancer specific				5-year BDDM			
**Category**	***P*-value**	**HR**	**95% CI**	**Category**	***P*-value**	**HR**	**95% CI**
Tumor size	1.67 × 10^-6^			Tumor size	9.89 × 10^-13^		
2 vs 1	0.0006	1.92	1.32 to 2.79	2 vs 1	1.73 × 10^-6^	2.4	1.68 to 3.44
3 vs 1	4.12 × 10^-6^	4.27	2.30 to 7.92	3 vs 1	2.38 × 10^-8^	5.21	2.92 to 9.30
4 vs 1	0.0001	3.80	1.95 to 7.41	4 vs 1	3.49 × 10^-11^	6.63	3.79 to 11.61
Nodal metastasis	1.70 × 10^-10^	3.57	2.41 to 5.27	Nodal metastasis	4.03 × 10^-10^	3.08	2.17 to 4.39
Distant metastasis	4.63 × 10^-10^	5.51	3.22 to 9.42				
progesterone receptor	0.0025	1.69	1.20 to 2.38	progesterone receptor	0.0398	1.39	1.02 to 1.91
Grade	2.12 × 10^-5^			Grade	0.0001		
2 vs 1	0.3528	1.31	0.74 to 2.31	2 vs 1	0.0111	2.07	1.18 to 3.63
3 vs 1	0.0004	2.76	1.57 to 4.86	3 vs 1	4.78 × 10^-5^	3.25	1.84 to 5.74
*PTEN *promoter variant	0.0119	2.01	1.17 to 3.46	*PTEN *promoter variant	0.0381	1.79	1.03 to 3.11

Models were built for 10-year breast cancer specific survival (left) and for 5-year breast cancer death or distant metastasis free survival (right). Factors included in the analysis were tumor size, nodal status, primary metastasis (for 10-year breast cancer specific only), estrogen receptor, progesterone receptor, Her2, p53, Ki67, and grade. Only the variables significant in the final step of the model are presented in the tables, demonstrating that carrying any of the *PTEN *promoter variants is an independent prognostic factor.

### Patients with *PTEN *promoter variants develop breast tumors with differential gene expression signature

**Figure 2:**
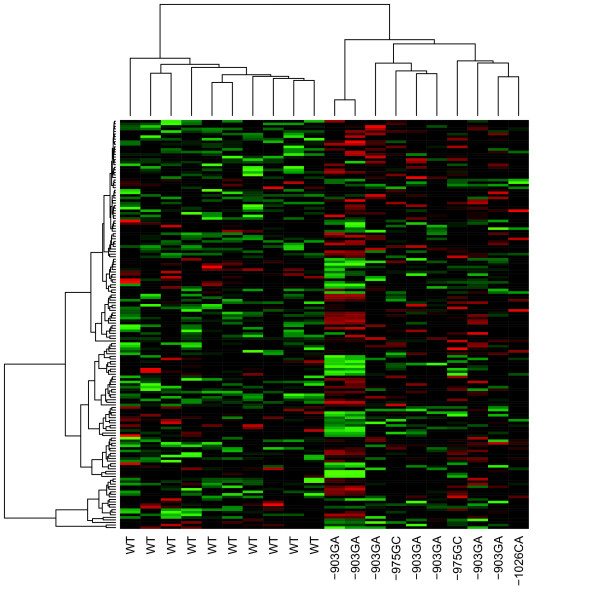
**The tumor gene expression signatures of the 160 differentially expressed genes**. The expression of the signature genes in the tumors of *PTEN *promoter variant carriers and non carriers show differential clustering separating the two groups.

We used microarray expression analysis to compare gene expression patterns in breast tumors from 10 patients with -903GA, -975GC, or -1026CA *PTEN *promoter variant and 10 matched non-carriers. A total of 160 genes were found to be differentially expressed (Additional file 2) with *P *< 0.01. No genes were significant after *post hoc *correction, hence we used a nominal *P*-value of 0.01 as the threshold of significance. Of the 160 genes, 104 genes were over-expressed and 56 under-expressed in the tumors of variant carriers. The differential expression between the subjects of two groups spanned between 1.81 and -1.85 base 2 log fold changes (3.5/-3.6 in natural scale). The samples successfully clustered using the expression profiles of the 160 differentially expressed genes as visualized in Figure 2, suggesting that this gene-set can be considered a signature for *PTEN *promoter variant carriers. The gene expression profiles of the tumors of different variant carriers co-segregated within the same branches of the hierarchical cluster. There was no segregation when the whole expression matrix (24,660 genes) was used in hierarchical clustering analysis. Differences in the expression levels of the *PTEN *gene could not be detected in microarray analysis between promoter variant carriers and non-carriers, possibly due to the limitations of the technology to detected small differences.

The differentially expressed genes were further annotated for their biological themes using DAVID functional annotation tools (http://david.abcc.ncifcrf.gov/) [44]. The genes up-regulated in the tumors of *PTEN *promoter variant carriers represented several biological families related to ATP-binding, protein phosphorylation and protein kinases. Likewise, functional groups of DNA-binding and transcription factor proteins were over-represented among the down-regulated genes (Additional file 3).

We compared the 160 differentially expressed genes with the gene expression signature of PTEN deficient tumors defined previously by Saal *et al. *[30]. The entities in the Saal signature list could be mapped to 151 unique Entrez Gene IDs. Of these, only one (*TUBB2C*, EG. 10383) was also found among the differentially expressed genes reported here. On the functional level, however, the Saal genes represented, among others, biological themes overlapping with those found here, such as phosphoprotein and ATP-binding from the up-regulated genes as well as the DNA-binding and transcription regulation from the down-regulated gene list.

### The gene signature of the *PTEN *promoter variant carrier tumors stratifies patients into two groups with different recurrence free survival

**Figure 3:**
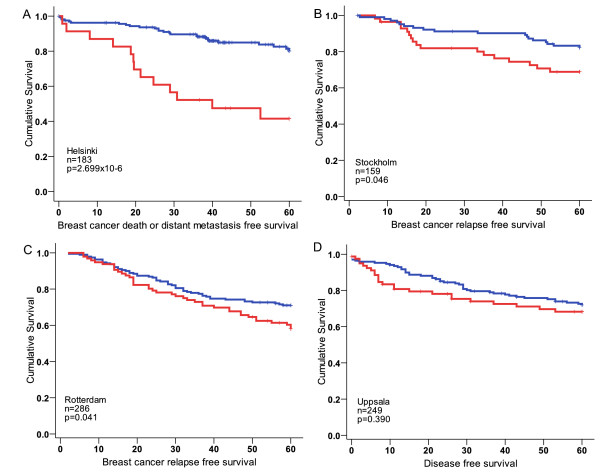
**Survival differences by the expression of the signature genes in independent breast cancer data sets**. Expression patterns of the 160 signature genes affect the five-year breast cancer recurrence in Helsinki breast cancer data set GSE24450 **(A) **as well as in other publicly available breast cancer gene expression data sets from Stockholm GSE1456 **(B) **and from Rotterdam GSE2034 **(C)**. Similar trend, although not significant was present also in Uppsala GSE4922 data set **(D)**.

We investigated the effects of the 160 signature genes on breast cancer survival and recurrence in a larger data set of 183 breast tumors (Helsinki data set) and in three independent publicly available breast cancer gene expression data sets with survival information from Sweden (GSE1456 [45] and GSE4922 [46]) and from the Netherlands (GSE2034 [47]). For all the data sets analyzed, the expression patterns for the signature genes were retrieved from the larger expression matrix and unsupervised segregative clustering (k-means) was used to assign the samples to two groups, which were then compared by log-rank test. The survival curves were visualized in Kaplan-Meier plots. The two groups of patients defined according to the expression of the 160 genes had distinct BDDM survival in the Helsinki data set (Figure 3A) (*P *= 2.699 × 10^-6^). The survival effect was further confirmed in two independent data sets (Figure 3B, C), and while the trend of the effect could be seen also in the third data set, the difference did not reach a statistically significant level (Figure 3D). We further applied the same approach to see if the gene expression signature of somatic loss of PTEN expression defined by Saal *et al. *[30] would have similar survival effects. The Saal signature also divided patients into two survival groups in all data sets with LogRank *P*-values in the Helsinki set *P *= 1.06 × 10^-6^, in the Stockholm set *P *= 0.003, in the Rotterdam set *P *= 0.001, and in the Uppsala set *P *= 0.002. In these data sets, 93%, 74%, 69% and 76% of the tumors, respectively, clustered in corresponding survival groups as those in the analysis by our *PTEN *promoter variant signature.

## Discussion

Here we have investigated germline genetic variation in the promoter region of the *PTEN *gene for breast cancer patient survival, with further gene expression analysis of breast tumors from the variant carriers. The -903GA, -975GC and -1026CA promoter variants were found to predict poor survival of breast cancer patients. In multivariate analysis adjusted for conventional prognostic factors, carrying any of the three promoter variants was an independent predictor of poor prognosis and approximately doubled the risk of the patients for distant metastasis or death within the follow-up time. One of the variants, -975GC, was also found to associate with having distant metastasis already at the time of diagnosis, and the -1026CA was found to be associated with increased proliferation of the tumor cells. These results suggest that the *PTEN *promoter variation has an effect on increased metastatic potential and progression of the tumor.

All the variants detected lie on the promoter region on nucleotides evolutionarily conserved in higher mammals (Additional file 4) and may affect the PTEN expression, although other functional mechanisms of the variants cannot be excluded at this phase. The *PTEN *gene expression levels in the variant carrier tumors were comparable to those in the non-carriers, possibly due to the limitations of the technology to detect small differences. This is consistent with the recent findings showing that even a subtle decrease in PTEN dose increased cancer susceptibility in mice, affected the cellular proliferation particularly in mouse mammary tissue, and altered the expression of proliferation related genes [31]. Indeed, the -1026CA variant associated significantly with proliferation rate of the tumors. Moreover, considering the role of *PTEN *as a dosage dependent tumor suppressor [48], even subtle changes in the expression of the *PTEN *gene might play a role in the very early stages of tumorigenesis. However, further studies will be needed to determine the functional effects of the variants.

The gene expression analysis showed 160 genes being differentially expressed in the tumors of the variant carriers compared to non-carrier tumors, with similar and indistinguishable patterns in the tumors of the patients with -903GA, -975GC or -1026CA variant. The up-regulated genes annotated in large proportion to entities related to ATP or nucleoside binding and phosphorylation while DNA binding and transcriptional functions were common among down-regulated genes (Supplementary Table S4). The most strongly down-regulated gene in the signature was interestingly *BAMBI *(BMP and Activin Membrane-Bound Inhibitor) (Supplementary Table S3). High expression of *BAMBI *has been shown to predict metastatic potential in colorectal cancer [49] and it is epigenetically silenced in high grade bladder carcinomas [50]. However, the signature represents a complex network of downstream effects of the *PTEN *promoter variants on tumor progression, with an overall effect on tumor phenotype and patient survival.

In the comparison of the differentially expressed genes found in this study and the somatic PTEN expression signature (by PTEN protein loss) in the study by Saal *et al. *[30] little overlap was seen at the single gene level. This was not surprising considering the profound differences in the microarray technologies used in the two studies, such as probe lengths and densities, as well as the laboratory procedures and analysis protocols, which together can make the comparison of the expression levels of individual genes in different studies challenging [51]. Furthermore, the changes in the expression of the *PTEN *gene caused by the inherited germ line variation could predispose the carriers already in early tumorigenesis to specific tumor progression pathways, whereas the Saal signature represents effects of somatic loss of PTEN expression at some later stage of tumor progression. However, the Saal gene list and the signature described here were overall functionally overlapping, representing similar gene functions.

The survival analysis of the 160-gene signature in the Helsinki expression data set of 183 breast tumors revealed a correlation with the disease recurrence. This effect was also confirmed in independent, publicly available breast cancer gene expression data sets. The goal of this analysis was to evaluate the potential of the signature genes in identifying groups of patients with different survival rates. In this analysis, some of the signature genes can be either up- or down-regulated in different patients. Hence, rather than evaluating the differential expression as such, the overall combinatorial effect of the signature genes needs to be evaluated. To achieve this, we applied the approach successfully used by Lukes *et al. *[52], in which the patients are divided into two groups by clustering analysis based on the overall expression of the signature genes. The survival differences between the two groups were then evaluated. Our results show that the expression signature of 160 genes associates with breast cancer prognosis in independent breast tumor gene expression data sets. These results emphasize the biological importance of the signature genes and their impact on breast cancer progression and show that the signature has a similar effect on breast cancer recurrence as the promoter variants. Indeed, the metastasis susceptibility effect by the germline variants is likely mediated by their downstream effects on the signature genes. Furthermore, when the gene signature of somatically aberrant PTEN expression by Saal *et al. *was used for the survival analysis, we found the clustering of the tumors into corresponding survival groups in each data set was highly correlated between the two signatures. This highlights the biological similarity of the two signatures and overall supports the inherited promoter variant's effect on the PTEN mediated tumor suppression.

## Conclusions

The *PTEN *gene and the AKT/PI3K pathway are among the most intensively studied targets in cancer research. So far, no associations of germline genetic variation in *PTEN *have been shown to exist with clinically relevant features outside hamartomatous polyposis syndromes. The genetic analyses together with gene expression analysis in this study suggest that inherited genetic variation in the *PTEN *promoter region affect the metastatic potential and tumor progression as well as gene expression profile in breast cancer, also with clinical implications for reduced survival of breast cancer patients. These findings also further strengthen the proposed role of germline variation on gene expression signatures and on metastatic potential of the tumors [52]. Further studies are warranted to establish the *PTEN *promoter variants as clinical markers for prognosis in breast cancer.

## Abbreviations

BDDM: breast cancer death or distant metastasis free survival; CISH: chromogenic *in situ *hybridization; CSGE: conformation sensitive gel electrophoresis; DGGE: denaturing gradient gel electrophoresis; ER: estrogen receptor; HR: hazard ratio; OR: odds ratio; PI3K: phosphatidylinositol-3-kinase; PR: progesterone receptor; *PTEN*: Phosphatase and tensin homolog; PTT: protein truncation test

## Competing interests

The authors declare that they have no competing interests.

## Authors' contributions

TH, DG and HN conceived and designed the study. TH and LMP performed the experiments. TH and DG analyzed the data and interpreted the results with HN. PH, CB and KA provided the study material and patient information. TH, DG and HN wrote the manuscript. All authors contributed to and approved the final manuscript.

## Supplementary Material

Additional file 1 **Table S1**. Associations of the PTEN promoter variants on tumor characteristics. Click here for file

Additional file 2 **Table S2**. A list of 160 differentially expressed genes in tumors of *PTEN *promoter variant carriers and matched non-carriers. Click here for file

Additional file 3 **Table S3**. Functional annotations for up and down regulated genes in the tumors of *PTEN *promoter variant carriers and non carriers. Click here for file

Additional file 4 **Figure S1**. Evolutional conservation of the *PTEN *promoter variants in higher mammals. Click here for file

## References

[B1] Miki Y Swensen J Shattuck-Eidens D Futreal PA Harshman K Tavtigian S Liu Q Cochran C Bennett LM Ding W A strong candidate for the breast and ovarian cancer susceptibility gene BRCA1 Science 1994 266 66 71 10.1126/science.7545954 7545954

[B2] Wooster R Bignell G Lancaster J Swift S Seal S Mangion J Collins N Gregory S Gumbs C Micklem G Identification of the breast cancer susceptibility gene BRCA2 Nature 1995 378 789 792 10.1038/378789a0 8524414

[B3] Lee EH Park SK Park B Kim SW Lee MH Ahn SH Son BH Yoo KY Kang D KOHBRA Research Group Korean Breast Cancer Society Effect of BRCA1/2 mutation on short-term and long-term breast cancer survival: a systematic review and meta-analysis Breast Cancer Res Treat 2010 122 11 25 10.1007/s10549-010-0859-2 20376556

[B4] Heikkinen T Karkkainen H Aaltonen K Milne RL Heikkila P Aittomaki K Blomqvist C Nevanlinna H The breast cancer susceptibility mutation PALB2 1592delT is associated with an aggressive tumor phenotype Clin Cancer Res 2009 15 3214 3222 10.1158/1078-0432.CCR-08-3128 19383810

[B5] Malkin D Li FP Strong LC Fraumeni JF Jr Nelson CE Kim DH Kassel J Gryka MA Bischoff FZ Tainsky MA Germ line p53 mutations in a familial syndrome of breast cancer, sarcomas, and other neoplasms Science 1990 250 1233 1238 10.1126/science.1978757 1978757

[B6] Tommiska J Eerola H Heinonen M Salonen L Kaare M Tallila J Ristimaki A von Smitten K Aittomaki K Heikkila P Blomqvist C Nevanlinna H Breast cancer patients with p53 Pro72 homozygous genotype have a poorer survival Clin Cancer Res 2005 11 5098 5103 10.1158/1078-0432.CCR-05-0173 16033823

[B7] Schmidt MK Tommiska J Broeks A van Leeuwen FE Van't Veer LJ Pharoah PD Easton DF Shah M Humphreys M Dork T Reincke SA Fagerholm R Blomqvist C Nevanlinna H Combined effects of single nucleotide polymorphisms TP53 R72P and MDM2 SNP309, and p53 expression on survival of breast cancer patients Breast Cancer Res 2009 11 R89 10.1186/bcr2460 20021639 PMC2815553

[B8] Nelen MR Padberg GW Peeters EA Lin AY van den Helm B Frants RR Coulon V Goldstein AM van Reen MM Easton DF Eeles RA Hodgsen S Mulvihill JJ Murday VA Tucker MA Mariman EC Starink TM Ponder BA Ropers HH Kremer H Longy M Eng C Localization of the gene for Cowden disease to chromosome 10q22-23 Nat Genet 1996 13 114 116 10.1038/ng0596-114 8673088

[B9] Liaw D Marsh DJ Li J Dahia PL Wang SI Zheng Z Bose S Call KM Tsou HC Peacocke M Eng C Parsons R Germline mutations of the PTEN gene in Cowden disease, an inherited breast and thyroid cancer syndrome Nat Genet 1997 16 64 67 10.1038/ng0597-64 9140396

[B10] Eng C Constipation, polyps, or cancer? Let PTEN predict your future Am J Med Genet A 2003 122A 315 322 10.1002/ajmg.a.20477 14518069

[B11] Teresi RE Zbuk KM Pezzolesi MG Waite KA Eng C Cowden syndrome-affected patients with PTEN promoter mutations demonstrate abnormal protein translation Am J Hum Genet 2007 81 756 767 10.1086/521051 17847000 PMC2227925

[B12] Zhou XP Waite KA Pilarski R Hampel H Fernandez MJ Bos C Dasouki M Feldman GL Greenberg LA Ivanovich J Matloff E Patterson A Pierpont ME Russo D Nassif NT Eng C Germline PTEN promoter mutations and deletions in Cowden/Bannayan-Riley-Ruvalcaba syndrome result in aberrant PTEN protein and dysregulation of the phosphoinositol-3-kinase/Akt pathway Am J Hum Genet 2003 73 404 411 10.1086/377109 12844284 PMC1180378

[B13] Stambolic V MacPherson D Sas D Lin Y Snow B Jang Y Benchimol S Mak TW Regulation of PTEN transcription by p53 Mol Cell 2001 8 317 325 10.1016/S1097-2765(01)00323-9 11545734

[B14] Sheng X Koul D Liu JL Liu TJ Yung WK Promoter analysis of tumor suppressor gene PTEN: identification of minimum promoter region Biochem Biophys Res Commun 2002 292 422 426 10.1006/bbrc.2002.6662 11906179

[B15] Carroll BT Couch FJ Rebbeck TR Weber BL Polymorphisms in PTEN in breast cancer families J Med Genet 1999 36 94 96 10051004 PMC1734307

[B16] Haiman CA Stram DO Cheng I Giorgi EE Pooler L Penney K Le Marchand L Henderson BE Freedman ML Common genetic variation at PTEN and risk of sporadic breast and prostate cancer Cancer Epidemiol Biomarkers Prev 2006 15 1021 1025 10.1158/1055-9965.EPI-05-0896 16702386

[B17] Guenard F Labrie Y Ouellette G Beauparlant CJ Bessette P Chiquette J Laframboise R Lepine J Lesperance B Pichette R Plante M Durocher F INHERIT BRCAs Germline mutations in the breast cancer susceptibility gene PTEN are rare in high-risk non-BRCA1/2 French Canadian breast cancer families Fam Cancer 2007 6 483 490 10.1007/s10689-007-9151-y 17636424

[B18] Li J Yen C Liaw D Podsypanina K Bose S Wang SI Puc J Miliaresis C Rodgers L McCombie R Bigner SH Giovanella BC Ittmann M Tycko B Hibshoosh H Wigler MH Parsons R PTEN, a putative protein tyrosine phosphatase gene mutated in human brain, breast, and prostate cancer Science 1997 275 1943 1947 10.1126/science.275.5308.1943 9072974

[B19] Steck PA Pershouse MA Jasser SA Yung WK Lin H Ligon AH Langford LA Baumgard ML Hattier T Davis T Frye C Hu R Swedlund B Teng DH Tavtigian SV Identification of a candidate tumour suppressor gene, MMAC1, at chromosome 10q23.3 that is mutated in multiple advanced cancers Nat Genet 1997 15 356 362 10.1038/ng0497-356 9090379

[B20] Cantley LC Neel BG New insights into tumor suppression: PTEN suppresses tumor formation by restraining the phosphoinositide 3-kinase/AKT pathway Proc Natl Acad Sci USA 1999 96 4240 4245 10.1073/pnas.96.8.4240 10200246 PMC33561

[B21] Shaw RJ Cantley LC Ras, PI(3)K and mTOR signalling controls tumour cell growth Nature 2006 441 424 430 10.1038/nature04869 16724053

[B22] Salmena L Carracedo A Pandolfi PP Tenets of PTEN tumor suppression Cell 2008 133 403 414 10.1016/j.cell.2008.04.013 18455982

[B23] Perren A Weng LP Boag AH Ziebold U Thakore K Dahia PL Komminoth P Lees JA Mulligan LM Mutter GL Eng C Immunohistochemical evidence of loss of PTEN expression in primary ductal adenocarcinomas of the breast Am J Pathol 1999 155 1253 1260 10.1016/S0002-9440(10)65227-3 10514407 PMC1867038

[B24] Depowski PL Rosenthal SI Ross JS Loss of expression of the PTEN gene protein product is associated with poor outcome in breast cancer Mod Pathol 2001 14 672 676 10.1038/modpathol.3880371 11454999

[B25] Bose S Crane A Hibshoosh H Mansukhani M Sandweis L Parsons R Reduced expression of PTEN correlates with breast cancer progression Hum Pathol 2002 33 405 409 10.1053/hupa.2002.124721 12055674

[B26] Tsutsui S Inoue H Yasuda K Suzuki K Higashi H Era S Mori M Reduced expression of PTEN protein and its prognostic implications in invasive ductal carcinoma of the breast Oncology 2005 68 398 404 10.1159/000086981 16020969

[B27] Perez-Tenorio G Alkhori L Olsson B Waltersson MA Nordenskjold B Rutqvist LE Skoog L Stal O PIK3CA mutations and PTEN loss correlate with similar prognostic factors and are not mutually exclusive in breast cancer Clin Cancer Res 2007 13 3577 3584 10.1158/1078-0432.CCR-06-1609 17575221

[B28] Lopez-Knowles E O'Toole SA McNeil CM Millar EK Qiu MR Crea P Daly RJ Musgrove EA Sutherland RL PI3K pathway activation in breast cancer is associated with the basal-like phenotype and cancer-specific mortality Int J Cancer 2010 126 1121 1131 19685490 10.1002/ijc.24831

[B29] Saal LH Gruvberger-Saal SK Persson C Lovgren K Jumppanen M Staaf J Jonsson G Pires MM Maurer M Holm K Koujak S Subramaniyam S Vallon-Christersson J Olsson H Su T Memeo L Ludwig T Ethier SP Krogh M Szabolcs M Murty VV Isola J Hibshoosh H Parsons R Borg A Recurrent gross mutations of the PTEN tumor suppressor gene in breast cancers with deficient DSB repair Nat Genet 2008 40 102 107 10.1038/ng.2007.39 18066063 PMC3018354

[B30] Saal LH Johansson P Holm K Gruvberger-Saal SK She QB Maurer M Koujak S Ferrando AA Malmstrom P Memeo L Isola J Bendahl PO Rosen N Hibshoosh H Ringner M Borg A Parsons R Poor prognosis in carcinoma is associated with a gene expression signature of aberrant PTEN tumor suppressor pathway activity Proc Natl Acad Sci USA 2007 104 7564 7569 10.1073/pnas.0702507104 17452630 PMC1855070

[B31] Alimonti A Carracedo A Clohessy JG Trotman LC Nardella C Egia A Salmena L Sampieri K Haveman WJ Brogi E Richardson AL Zhang J Pandolfi PP Subtle variations in Pten dose determine cancer susceptibility Nat Genet 2010 42 454 458 10.1038/ng.556 20400965 PMC3118559

[B32] Syrjakoski K Vahteristo P Eerola H Tamminen A Kivinummi K Sarantaus L Holli K Blomqvist C Kallioniemi OP Kainu T Nevanlinna H Population-based study of BRCA1 and BRCA2 mutations in 1035 unselected Finnish breast cancer patients J Natl Cancer Inst 2000 92 1529 1531 10.1093/jnci/92.18.1529 10995809

[B33] Kilpivaara O Bartkova J Eerola H Syrjakoski K Vahteristo P Lukas J Blomqvist C Holli K Heikkila P Sauter G Kallioniemi OP Bartek J Nevanlinna H Correlation of CHEK2 protein expression and c.1100delC mutation status with tumor characteristics among unselected breast cancer patients Int J Cancer 2005 113 575 580 10.1002/ijc.20638 15472904

[B34] Fagerholm R Hofstetter B Tommiska J Aaltonen K Vrtel R Syrjakoski K Kallioniemi A Kilpivaara O Mannermaa A Kosma VM Uusitupa M Eskelinen M Kataja V Aittomaki K von Smitten K Heikkila P Lukas J Holli K Bartkova J Blomqvist C Bartek J Nevanlinna H NAD(P)H:quinone oxidoreductase 1 NQO1*2 genotype (P187S) is a strong prognostic and predictive factor in breast cancer Nat Genet 2008 40 844 853 10.1038/ng.155 18511948

[B35] Vahteristo P Eerola H Tamminen A Blomqvist C Nevanlinna H A probability model for predicting BRCA1 and BRCA2 mutations in breast and breast-ovarian cancer families Br J Cancer 2001 84 704 708 10.1054/bjoc.2000.1626 11237395 PMC2363799

[B36] Eerola H Heikkila P Tamminen A Aittomaki K Blomqvist C Nevanlinna H Histopathological features of breast tumours in BRCA1, BRCA2 and mutation-negative breast cancer families Breast Cancer Res 2005 7 R93 100 10.1186/bcr953 15642173 PMC1064101

[B37] Elston CW Ellis IO Pathological prognostic factors in breast cancer. I. The value of histological grade in breast cancer: experience from a large study with long-term follow-up Histopathology 1991 19 403 410 10.1111/j.1365-2559.1991.tb00229.x 1757079

[B38] Tommiska J Bartkova J Heinonen M Hautala L Kilpivaara O Eerola H Aittomaki K Hofstetter B Lukas J von Smitten K Blomqvist C Ristimaki A Heikkila P Bartek J Nevanlinna H The DNA damage signalling kinase ATM is aberrantly reduced or lost in BRCA1/BRCA2-deficient and ER/PR/ERBB2-triple-negative breast cancer Oncogene 2008 27 2501 2506 10.1038/sj.onc.1210885 17982490

[B39] Ahlin C Aaltonen K Amini RM Nevanlinna H Fjallskog ML Blomqvist C Ki67 and cyclin A as prognostic factors in early breast cancer. What are the optimal cut-off values? Histopathology 2007 51 491 498 10.1111/j.1365-2559.2007.02798.x 17711446

[B40] Gentleman RC Carey VJ Bates DM Bolstad B Dettling M Dudoit S Ellis B Gautier L Ge Y Gentry J Hornik K Hothorn T Huber W Iacus S Irizarry R Leisch F Li C Maechler M Rossini AJ Sawitzki G Smith C Smyth G Tierney L Yang JY Zhang J Bioconductor: open software development for computational biology and bioinformatics Genome Biol 2004 5 R80 10.1186/gb-2004-5-10-r80 15461798 PMC545600

[B41] Du P Kibbe WA Lin SM lumi: a pipeline for processing Illumina microarray Bioinformatics 2008 24 1547 1548 10.1093/bioinformatics/btn224 18467348

[B42] Bolstad BM Irizarry RA Astrand M Speed TP A comparison of normalization methods for high density oligonucleotide array data based on variance and bias Bioinformatics 2003 19 185 193 10.1093/bioinformatics/19.2.185 12538238

[B43] Smyth GK Gentleman R, Carey V, Dudoit S, Irizarry WH R Limma: linear models for microarray data Bioinformatics and Computational Biology Solutions Using R and Bioconductor 2005 New York: Springer 397 420

[B44] Huang da W Sherman BT Lempicki RA Systematic and integrative analysis of large gene lists using DAVID bioinformatics resources Nat Protoc 2009 4 44 57 19131956 10.1038/nprot.2008.211

[B45] Pawitan Y Bjohle J Amler L Borg AL Egyhazi S Hall P Han X Holmberg L Huang F Klaar S Liu ET Miller L Nordgren H Ploner A Sandelin K Shaw PM Smeds J Skoog L Wedren S Bergh J Gene expression profiling spares early breast cancer patients from adjuvant therapy: derived and validated in two population-based cohorts Breast Cancer Res 2005 7 R953 964 10.1186/bcr1325 16280042 PMC1410752

[B46] Ivshina AV George J Senko O Mow B Putti TC Smeds J Lindahl T Pawitan Y Hall P Nordgren H Wong JE Liu ET Bergh J Kuznetsov VA Miller LD Genetic reclassification of histologic grade delineates new clinical subtypes of breast cancer Cancer Res 2006 66 10292 10301 10.1158/0008-5472.CAN-05-4414 17079448

[B47] Wang Y Klijn JG Zhang Y Sieuwerts AM Look MP Yang F Talantov D Timmermans M Meijer-van Gelder ME Yu J Jatkoe T Berns EM Atkins D Foekens JA Gene-expression profiles to predict distant metastasis of lymph-node-negative primary breast cancer Lancet 2005 365 671 679 15721472 10.1016/S0140-6736(05)17947-1

[B48] Carracedo A Alimonti A Pandolfi PP PTEN level in tumor suppression: How much is too little? Cancer Res 2011 17 629 633 21266353 10.1158/0008-5472.CAN-10-2488 PMC3249925

[B49] Fritzmann J Morkel M Besser D Budczies J Kosel F Brembeck FH Stein U Fichtner I Schlag PM Birchmeier W A colorectal cancer expression profile that includes transforming growth factor beta inhibitor BAMBI predicts metastatic potential Gastroenterology 2009 137 165 175 10.1053/j.gastro.2009.03.041 19328798

[B50] Khin SS Kitazawa R Win N Aye TT Mori K Kondo T Kitazawa S BAMBI gene is epigenetically silenced in subset of high-grade bladder cancer Int J Cancer 2009 125 328 338 10.1002/ijc.24318 19326429

[B51] Draghici S Khatri P Eklund AC Szallasi Z Reliability and reproducibility issues in DNA microarray measurements Trends Genet 2006 22 101 109 10.1016/j.tig.2005.12.005 16380191 PMC2386979

[B52] Lukes L Crawford NP Walker R Hunter KW The origins of breast cancer prognostic gene expression profiles Cancer Res 2009 69 310 318 10.1158/0008-5472.CAN-08-3520 19118016 PMC2613551

