# Supplementary material for: Variants on the promoter region of PTEN affect breast cancer progression and patient survival
Source: Breast Cancer Res. 2011 Dec 15;13(6):R130. doi: 10.1186/bcr3076 (PMC3326572; doi:10.1186/bcr3076)
Supplement: Additional file 1 — Table S1. Associations of the PTEN promoter variants on tumor characteristics. [file bcr3076-S1.PDF]

Supplementary Table S2. Associations of the PTEN promoter variants on tumor characteristics

			PTEN -903GA							PTEN -975GC							PTEN -1026CA						
Category	Total	%	GG	%	GA	%	Sig.	OR	95% CI	GG	%	GC	%	Sig.	OR	95% CI	CC	%	CA	%	Sig.	OR	95% CI
T																							
1	1457	61.4%	1397	61.7%	34	53.1%	0.1657	1.42	0.86-2.34	1416	61.5%	15	57.7%	0.6928	1.17	0.54-2.56	1406	61.3%	29	67.4%	0.4117	0.76	0.40-1.45
2	749	31.6%	711	31.4%	23	35.9%	1 vs 2,3,4			726	31.5%	8	30.8%	1 vs 2,3,4			724	31.6%	13	30.2%	1 vs 2,3,4		
3	81	3.4%	76	3.4%	3	4.7%				79	3.4%	0	0.0%				80	3.5%	0	0.0%			
4	86	3.6%	81	3.6%	4	6.3%				82	3.6%	3	11.5%				84	3.7%	1	2.3%			
N																							
negative	1307	55.4%	1251	55.6%	29	44.6%	0.0798	1.55	0.95-2.55	1269	55.4%	11	44.0%	0.2546	1.58	0.71-3.50	1263	55.4%	21	48.8%	0.3933	1.30	0.71-2.38
positive	1053	44.6%	1000	44.4%	36	55.4%				1022	44.6%	14	56.0%				1018	44.6%	22	51.2%			
M																							
negative	2304	96.5%	2198	96.4%	64	98.5%	0.7267	0.42	0.06-3.09	2239	96.6%	23	85.2%	0.0132	4.99	1.69-14.78	2229	96.5%	41	97.6%	1.0000	0.67	0.09-4.94
positive	83	3.5%	81	3.6%	1	1.5%				78	3.4%	4	14.8%				81	3.5%	1	2.4%			
Grade																							
1	588	25.8%	565	26.0%	17	27.4%	0.3758	1.27	0.75-2.17	576	26.1%	6	23.1%	0.8277	1.10	0.47-2.54	569	25.8%	9	22.5%	0.2280	1.48	0.78-2.84
2	1032	45.3%	985	45.3%	24	38.7%	1 and 2 vs 3			997	45.1%	12	46.2%	1 and 2 vs 3			1000	45.4%	16	40.0%	1 and 2 vs 3		
3	656	28.8%	624	28.7%	21	33.9%				637	28.8%	8	30.8%				634	28.8%	15	37.5%			
Tumor histology																							
Ductal	1615	67.3%	1541	67.2%	45	69.2%	0.7351	0.91	0.53-1.56	1565	67.2%	21	77.8%	0.2426	0.58	0.23-1.45	1555	67.0%	33	76.7%	0.1762	0.61	0.30-1.25
Lobular	506	21.1%	484	21.1%	15	23.1%	Ductal vs any other			495	21.2%	4	14.8%	Ductal vs any other			494	21.3%	6	14.0%	Ductal vs any other		
Medullary	32	1.3%	31	1.4%	0	0.0%				31	1.3%	0	0.0%				31	1.3%	0	0.0%			
other	248	10.3%	236	10.3%	5	7.7%				239	10.3%	2	7.4%				242	10.4%	4	9.3%			
ER																							
negative	434	19.0%	411	18.9%	16	25.4%	0.1948	0.68	0.38-1.22	425	19.2%	2	8.0%	0.1565	2.73	0.64-11.63	418	18.9%	11	26.2%	0.2361	0.66	0.33-1.32
positive	1847	81.0%	1765	81.1%	47	74.6%				1789	80.8%	23	92.0%				1789	81.1%	31	73.8%			
PR																							
negative	792	34.8%	749	34.5%	27	42.9%	0.1671	0.70	0.42-1.16	769	34.8%	7	28.0%	0.4798	1.37	0.57-3.30	764	34.6%	17	40.5%	0.4321	0.78	0.42-1.45
positive	1487	65.2%	1425	65.5%	36	57.1%				1443	65.2%	18	72.0%				1441	65.4%	25	59.5%			
HER2																							
negative	1142	85.2%	1088	85.3%	24	77.4%	0.2256	1.69	0.72-3.97	1100	85.1%	12	85.7%	0.9466	0.95	0.21-4.28	1099	85.1%	20	87.0%	0.8004	0.85	0.25-2.90
positive	198	14.8%	188	14.7%	7	22.6%				193	14.9%	2	14.3%				193	14.9%	3	13.0%			
p53																							
negative	1035	79.7%	984	79.5%	26	81.3%	0.8135	0.90	0.37-2.20	1000	79.6%	10	76.9%	0.8105	1.17	0.32-4.29	998	79.6%	19	82.6%	0.7212	0.82	0.28-2.43
positive	264	20.3%	253	20.5%	6	18.8%				256	20.4%	3	23.1%				256	20.4%	4	17.4%			
Ki67																							
0	441	20.8%	417	20.6%	13	23.6%	0.0826	1.68	0.93-3.04	422	20.6%	8	32.0%	0.9874	1.01	0.38-2.70	419	20.4%	13	32.5%	0.0154	2.21	1.15-4.28
1	861	40.6%	829	41.0%	19	34.5%	0,1,2 vs 3			840	40.9%	8	32.0%	0,1,2 vs 3			838	40.9%	12	30.0%	0,1,2 vs 3		
2	396	18.7%	380	18.8%	7	12.7%				383	18.7%	4	16.0%				391	19.1%	1	2.5%			
3	423	19.9%	397	19.6%	16	29.1%				408	19.9%	5	20.0%				401	19.6%	14	35.0%			
