# Supplementary material for: Variants on the promoter region of PTEN affect breast cancer progression and patient survival
Source: Breast Cancer Res. 2011 Dec 15;13(6):R130. doi: 10.1186/bcr3076 (PMC3326572; doi:10.1186/bcr3076)
Supplement: Additional file 2 — Table S2. A list of 160 differentially expressed genes in tumors of PTEN promoter variant carriers and matched non-carriers. [file bcr3076-S2.PDF]

Supplementary Table S3. A list of 160 differentially expressed genes in tumors of *PTEN* promoter variant carriers and matched non-carriers, with differential expression (logFC) and p-values.

Entrez Gene ID	Gene Symbol	logFC	p-value	Entrez Gene ID	Gene Symbol	logFC	p-value
374897	SBSN	1.8194	0.0030	648133	-	-0.1267	0.0088
56675	NRIP3	1.3061	0.0027	6998	TDGF3	-0.1334	0.0064
51099	ABHD5	0.8903	0.0019	170825	GSX2	-0.1543	0.0036
2817	GPC1	0.8537	0.0057	390999	PRAMEF12	-0.1566	0.0017
9209	LRRFIP2	0.8510	0.0007	286205	SCAI	-0.1649	0.0045
3638	INSIG1	0.7982	0.0019	5539	PPY	-0.1720	0.0057
9903	KLHL21	0.7956	0.0012	2104	ESRRG	-0.1866	0.0024
79614	C5orf23	0.7576	0.0009	91522	COL23A1	-0.1910	0.0086
23466	CBX6	0.7226	0.0048	10044	SH2D3C	-0.1945	0.0035
6744	SSFA2	0.6613	0.0013	115560	ZNF501	-0.2214	0.0057
8853	ASAP2	0.6552	0.0005	728308	-	-0.2317	0.0031
5714	PSMD8	0.6497	0.0064	56961	SHD	-0.2421	0.0062
79669	C3orf52	0.6443	0.0076	10170	DHRS9	-0.2581	0.0041
7866	IFRD2	0.6150	0.0020	92797	HELB	-0.2588	0.0059
6988	TCTA	0.5774	0.0001	645899	RPS28P4	-0.2620	0.0051
10352	WARS2	0.5771	0.0015	285176	RPL10P6	-0.2624	0.0005
3475	IFRD1	0.5707	0.0050	80034	CSRNP3	-0.2642	0.0032
10039	PARP3	0.5679	0.0041	23130	ATG2A	-0.2705	0.0080
10383	TUBB2C	0.5675	0.0043	63915	MUTED	-0.2776	0.0076
643319	-	0.5556	0.0075	54461	FBXW5	-0.2916	0.0058
7384	UQCRC1	0.5546	0.0011	89822	KCNK17	-0.3126	0.0079
55129	ANO10	0.5532	0.0013	2494	NR5A2	-0.3367	0.0013
284023	LOC284023	0.5481	0.0059	22929	SEPHS1	-0.3740	0.0085
9861	PSMD6	0.5296	0.0051	2073	ERCC5	-0.3796	0.0040
7873	MANF	0.5262	0.0097	5830	PEX5	-0.3959	0.0061
80145	THOC7	0.5074	0.0009	9365	KL	-0.3963	0.0035
54606	DDX56	0.5024	0.0096	1998	ELF2	-0.4205	0.0052
9943	OXSR1	0.4973	0.0039	5775	PTPN4	-0.4223	0.0020
5045	FURIN	0.4858	0.0067	84701	COX4I2	-0.4348	0.0087
54942	C9orf6	0.4849	0.0054	23047	PDS5B	-0.4475	0.0011
7165	TPD52L2	0.4838	0.0081	9937	DCLRE1A	-0.4555	0.0073
5372	PMM1	0.4829	0.0089	56987	BBX	-0.4607	0.0025
908	CCT6A	0.4796	0.0036	9651	PLCH2	-0.4806	0.0028
8668	EIF3I	0.4778	0.0049	1777	DNASE2	-0.4886	0.0093
1454	CSNK1E	0.4665	0.0072	54464	XRN1	-0.4922	0.0024
10425	ARIH2	0.4642	0.0035	23144	ZC3H3	-0.5242	0.0015
23016	EXOSC7	0.4568	0.0048	64881	PCDH20	-0.5298	0.0003
11070	TMEM115	0.4479	0.0017	23240	KIAA0922	-0.5477	0.0024
6390	SDHB	0.4384	0.0078	10559	SLC35A1	-0.5526	0.0026
22907	DHX30	0.4312	0.0014	54941	RNF125	-0.5618	0.0047
51447	IP6K2	0.4278	0.0065	54996	MOSC2	-0.5682	0.0019
51619	UBE2D4	0.4233	0.0017	4084	MXD1	-0.5729	0.0090
11099	PTPN21	0.4231	0.0076	1936	EEF1D	-0.5963	0.0031
201626	PDE12	0.4228	0.0028	79622	SNRNP25	-0.6303	0.0080
51460	SFMBT1	0.4197	0.0097	10867	TSPAN9	-0.6365	0.0099
23276	KLHL18	0.4130	0.0025	65084	TMEM135	-0.6656	0.0093
26097	C1orf77	0.4127	0.0071	9580	SOX13	-0.7295	0.0038
1605	DAG1	0.4078	0.0082	57419	SLC24A3	-0.8620	0.0018
7532	YWHAG	0.4027	0.0004	22882	ZHX2	-0.9829	0.0008

7584	ZNF35	0.4000	0.0047	79805	VASH2	-0.9983	0.0018
1781	DYNC1I2	0.3970	0.0020	2982	GUCY1A3	-1.1562	0.0078
9807	IP6K1	0.3759	0.0013	115572	FAM46B	-1.1805	0.0011
441408	-	0.3677	0.0087	187	APLNR	-1.2116	0.0061
5431	POLR2B	0.3586	0.0017	3215	HOXB5	-1.2866	0.0082
79714	CCDC51	0.3557	0.0036	30061	SLC40A1	-1.8039	0.0029
91289	LMF2	0.3538	0.0100	25805	BAMBI	-1.8561	0.0029
157922	CAMSAP1	0.3443	0.0054				
92399	MRRF	0.3408	0.0055				
170487	C20orf134	0.3379	0.0067				
25930	PTPN23	0.3301	0.0051				
339487	ZBTB8OS	0.3276	0.0026				
23604	DAPK2	0.3259	0.0060				
2130	EWSR1	0.3121	0.0065				
984	CDK11B	0.2737	0.0073				
652615	-	0.2561	0.0054				
641699	-	0.2549	0.0083				
644278	-	0.2494	0.0070				
7407	VARS	0.2438	0.0016				
3755	KCNG1	0.2359	0.0081				
388939	C2orf71	0.2313	0.0045				
65996	MGC2752	0.2048	0.0068				
643403	-	0.1961	0.0036				
644680	-	0.1951	0.0044				
147166	TRIM16L	0.1947	0.0021				
389118	CDHR4	0.1932	0.0034				
253128	C12orf33	0.1902	0.0049				
652565	-	0.1796	0.0019				
152831	KLB	0.1784	0.0087				
5884	RAD17	0.1740	0.0020				
391574	-	0.1704	0.0094				
285733	LOC285733	0.1676	0.0052				
732343	-	0.1629	0.0044				
649770	-	0.1627	0.0055				
645757	-	0.1601	0.0051				
643982	-	0.1541	0.0053				
644225	-	0.1517	0.0094				
84254	CAMKK1	0.1517	0.0048				
643912	-	0.1483	0.0054				
204474	PDILT	0.1440	0.0061				
390829	-	0.1411	0.0067				
651850	-	0.1411	0.0063				
403282	OR6C65	0.1344	0.0074				
79368	FCRL2	0.1335	0.0046				
154810	AMOTL1	0.1335	0.0035				
653882	LOC653882	0.1306	0.0083				
259232	NALCN	0.1258	0.0059				
729393	-	0.1232	0.0097				
643520	LOC643520	0.1177	0.0066				
58511	DNASE2B	0.1102	0.0067				
199720	GGN	0.1098	0.0090				
284656	EPHA10	0.1085	0.0090				
135152	B3GAT2	0.1081	0.0039				
84063	KIRREL2	0.0999	0.0097				
