# Supplementary material for: Variants on the promoter region of PTEN affect breast cancer progression and patient survival
Source: Breast Cancer Res. 2011 Dec 15;13(6):R130. doi: 10.1186/bcr3076 (PMC3326572; doi:10.1186/bcr3076)
Supplement: Additional file 3 — Table S3. Functional annotations for up and down regulated genes in the tumors of PTEN promoter variant carriers and non carriers. [file bcr3076-S3.PDF]

Supplementary Table S4. Functional annotations by DAVID functional annotations tools for up and down regulated genes in the tumors of *PTEN* promoter variant carriers and non carriers.

List of up regulated gene functional annotations

Category	Term	Count	%	p-value
SP_PIR_KEYWORDS	atp-binding	16	18.6	0.0004
GOTERM_MF_FAT	GO:0001882~nucleoside binding	17	19.8	0.0006
GOTERM_MF_FAT	GO:0005524~ATP binding	16	18.6	0.0008
GOTERM_MF_FAT	GO:0032559~adenyl ribonucleotide binding	16	18.6	0.0009
GOTERM_MF_FAT	GO:0030554~adenyl nucleotide binding	16	18.6	0.0015
SP_PIR_KEYWORDS	nucleotide-binding	17	19.8	0.0016
GOTERM_MF_FAT	GO:0001883~purine nucleoside binding	16	18.6	0.0017
GOTERM_MF_FAT	GO:0032555~purine ribonucleotide binding	17	19.8	0.0025
GOTERM_MF_FAT	GO:0032553~ribonucleotide binding	17	19.8	0.0025
GOTERM_BP_FAT	GO:0006796~phosphate metabolic process	12	14.0	0.0033
GOTERM_BP_FAT	GO:0006793~phosphorus metabolic process	12	14.0	0.0033
GOTERM_MF_FAT	GO:0017076~purine nucleotide binding	17	19.8	0.0039
GOTERM_MF_FAT	GO:0000166~nucleotide binding	18	20.9	0.0077
GOTERM_BP_FAT	GO:0016310~phosphorylation	10	11.6	0.0083
SP_PIR_KEYWORDS	kinase	9	10.5	0.0083
INTERPRO	IPR007701:Interferon-related developmental regulator, N-terminal	2	2.3	0.0085
INTERPRO	IPR006921:Interferon-related developmental regulator, C-terminal	2	2.3	0.0085
GOTERM_MF_FAT	GO:0004672~protein kinase activity	8	9.3	0.0123
INTERPRO	IPR017441:Protein kinase, ATP binding site	7	8.1	0.0129
SP_PIR_KEYWORDS	transferase	13	15.1	0.0135
UP_SEQ_FEATURE	domain:Protein kinase	7	8.1	0.0146
INTERPRO	IPR000719:Protein kinase, core	7	8.1	0.0158
UP_SEQ_FEATURE	compositionally biased region:Poly-Glu	7	8.1	0.0184
UP_SEQ_FEATURE	binding site:ATP	7	8.1	0.0275
GOTERM_MF_FAT	GO:0008440~inositol trisphosphate 3-kinase activity	2	2.3	0.0288
INTERPRO	IPR005522:Inositol polyphosphate kinase	2	2.3	0.0295
GOTERM_MF_FAT	GO:0051766~inositol trisphosphate kinase activity	2	2.3	0.0328
UP_SEQ_FEATURE	domain:Ig-like C2-type 4	3	3.5	0.0413
SP_PIR_KEYWORDS	protein biosynthesis	4	4.7	0.0450
UP_SEQ_FEATURE	nucleotide phosphate-binding region:ATP	9	10.5	0.0507
SP_PIR_KEYWORDS	phosphoprotein	39	45.3	0.0574
UP_SEQ_FEATURE	active site:Proton acceptor	7	8.1	0.0604
INTERPRO	IPR008271:Serine/threonine protein kinase, active site	5	5.8	0.0643
GOTERM_BP_FAT	GO:0046854~phosphoinositide phosphorylation	2	2.3	0.0645
SMART	SM00220:S_TKc	4	4.7	0.0668
INTERPRO	IPR017442:Serine/threonine protein kinase-related	5	5.8	0.0670
GOTERM_BP_FAT	GO:0046834~lipid phosphorylation	2	2.3	0.0687
GOTERM_BP_FAT	GO:0006468~protein amino acid phosphorylation	7	8.1	0.0743
SP_PIR_KEYWORDS	serine/threonine-protein kinase	5	5.8	0.0771
GOTERM_MF_FAT	GO:0004683~calmodulin-dependent protein kinase activity	2	2.3	0.0800
GOTERM_BP_FAT	GO:0034660~ncRNA metabolic process	4	4.7	0.0820
SP_PIR_KEYWORDS	calmodulin-binding	3	3.5	0.0910
UP_SEQ_FEATURE	domain:Ig-like C2-type 3	3	3.5	0.0946
GOTERM_BP_FAT	GO:0006650~glycerophospholipid metabolic process	3	3.5	0.0964
INTERPRO	IPR002290:Serine/threonine protein kinase	4	4.7	0.0985

List of down regulated genes functional annotations

Category	Term	Count	%	p-value
GOTERM_MF_FAT	GO:0003677~DNA binding	15	27.8	0.0034
SP_PIR_KEYWORDS	dna-binding	13	24.1	0.0035
GOTERM_MF_FAT	GO:0003700~transcription factor activity	9	16.7	0.0053
GOTERM_MF_FAT	GO:0043565~sequence-specific DNA binding	7	13.0	0.0069
SP_PIR_KEYWORDS	transcription regulation	12	22.2	0.0179
SP_PIR_KEYWORDS	Transcription	12	22.2	0.0207
GOTERM_MF_FAT	GO:0030528~transcription regulator activity	10	18.5	0.0233
GOTERM_CC_FAT	GO:0019898~extrinsic to membrane	5	9.3	0.0304
SP_PIR_KEYWORDS	nuclease	3	5.6	0.0334
GOTERM_BP_FAT	GO:0006350~transcription	12	22.2	0.0516
GOTERM_MF_FAT	GO:0004520~endodeoxyribonuclease activity	2	3.7	0.0609
GOTERM_MF_FAT	GO:0004518~nuclease activity	3	5.6	0.0743
GOTERM_MF_FAT	GO:0004536~deoxyribonuclease activity	2	3.7	0.0926
GOTERM_BP_FAT	GO:0045449~regulation of transcription	13	24.1	0.0936
