# Supplementary material for: Variants on the promoter region of PTEN affect breast cancer progression and patient survival
Source: Breast Cancer Res. 2011 Dec 15;13(6):R130. doi: 10.1186/bcr3076 (PMC3326572; doi:10.1186/bcr3076)
Supplement: Additional file 4 — Figure S1. Evolutional conservation of the PTEN promoter variants in higher mammals. [file bcr3076-S4.PDF]

```
Homo_sapiens >  chromosome:GRCh37:10:89623120:89623390:1
Pan_troglodytes >  chromosome:CHIMP2.1:10:88104300:88104571:1
Pongo_pygmaeus >  chromosome:PPYG2:10:86576060:86576326:-1
Macaca_mulatta >  chromosome:MMUL_1:9:87444480:87444747:1
Mus_musculus >  chromosome:NCBIM37:19:32831887:32832118:1
Bos_taurus >  chromosome:Btau_4.0:26:9876875:9877139:1
Canis_familiaris >  chromosome:BROADD2:26:40896602:40896832:1
```

```
                                                                                                 -1026CA
Homo_sapiens       TGCGGCTTGGGGACTCTGCGCTCGCACCCAGAGCTACCGCT-CTG--CCCCCT------CCTACCGCCCCCTGCCCTGCC-----CTGCC-CTCCCCTCGCCCGGCGCGGTCCCGTCCGC
Pan_troglodytes    TGCGGCTTGGGGGCTCTGCGCTCGCACCCAGAGCTATCGCT-CTA--CCCCCT------CCTACCGCCCCCTGCCCTACC-----CTGCC-CTCCCCTCGCCCGGCGCGGTCCCGTCCGC
Pongo_pygmaeus     TGCGGCTTGGGGGCTCTGCGCTCGCACCCAGAGCTACCGCT-CTG--CCCCCT------CCTACCTCCCCCTGCC----------CTGCC-CTCCCCTCGCCCGGCGCGGTCCCGTCCGC
Macaca_mulatta     TACGGCTTGGGGGCTTTGCGCTCGCACCCGGAGCTACCGCTCCTG--CCCCCG------CCTACCGCCCCCTGCC----------CTGCC-CTCCCCTCGCCCGGCACGGTCCAGTCCGC
Mus_musculus       ---------------------TC------------CCCGCCCCTG--TCCCTT------CCGACG---CCCCGCC----------CCGCCCCGCCCCGTCCCCGGCTCAG---CGCCCGC
Bos_taurus         TGCAGGTTGGGGACTCGGCGCTTGCACCCGGAGCTGCACCTTCTG--CCGCCTCCCTAT------AGTCTCGGCC-----CTGCCCTTCC-CTCCCCTCCCCTGGCGCGGT---------
Canis_familiaris   ----------------GGCGGTCGCCCCC-----CGCCCCTCCCC--CCGCCC------------GGCCCCGGCC-----CCCCCCGGCC-CCCGCCTCCCCCCGCCT------------
```

```
                                            -975GC                                                                  -903GA
Homo_sapiens       CTCTCGCTCGCCTCCCGCCTCCCCTC-GGTCTTCCGAGGCGCCCGG-GCTCCCGGCG-CGGCG---GCGGAGGGGGCGGGCAGGCCGGCG--GGCGGTGATGTGGCGGGACTCTTTATGC
Pan_troglodytes    CTCTCGCTCGCCTCCCGCCTCCCCTC-GGTCTCCCGAGGCGCCCGG-GCTCCCGGCG-CGGCG---GCGGAGGGGGCGGGCAGGCCGGCG--GGCGGTGATGTGGCGGGACTCTTTATGC
Pongo_pygmaeus     CTCTCGCTCGCCTCCCGCCTCCCCTC-GGTCTCCCGAGGCTCCCGG-GCTCCCAGCG-CGGCG---GCGGAGGGGGCGGGCAGGCCGGCG--GACGGTGATGTGGCGGGACTCTTTATGC
Macaca_mulatta     CTCTCGTTCGGCTCCCGCCTCCCCTC-GGTCTCCCGAGGAGCCCGG-GCTCCCGGCG-CGGCG---GCGGAGGGGGCGGGCAGGCCGGCG--GGCGGTGATGTGGCGGGACTCTTTATGC
Mus_musculus       CTCCCGCCCGCCTCCCGCCTCCCCTCCGGCTTTCCGAGGCGCCCTGCTCTCCCGGCG-GGGCG---GCGGAGGGGGCGGGCTGGCCGGCG--CACGGTGATGTGGCGGGACTCTTTGTGC
Bos_taurus         --CGGGTACGCCTCCCTCCTCCCCTC-GGGCTCCCGAGGCGCCTGG-ACTCCCGGCG-CGGCGGAGGCGGAGGGGGCGGGCAGGCCGGCG--GGCGGTGATGTGGCGGGACTCTTTGTGC
Canis_familiaris   --CCC-CCCGGCCCCCGCCTCCCCCC-GGGCTC-CGGGGCGCC-GG-GCTCGCGGCGGCGGCGGCGGCGGCGGCGGCGGGGGCGGGGGCGGCGGTGATGTGGCGGGACTCTTTGTGC
```
